# An Unexpected Complication Due to Metformin Use After Femur Fracture Operation: Metabolic Acidosis Without Lactic Acidosis

**DOI:** 10.7759/cureus.4584

**Published:** 2019-05-02

**Authors:** Eylem Yasar, Basak Altıparmak, Semra Gümüş Demirbilek

**Affiliations:** 1 Anesthesiology, Mugla Sıtkı Kocman University, Muğla, TUR; 2 Anesthesiology, Mugla Sitki Kocman University, Muğla, TUR

**Keywords:** metformin, methabolic acidosis, intensive care unit

## Abstract

A 74-year- old male, who was known to have hypertension, chronic obstructive lung disease, and benign prostate hyperplasia, was evaluated preoperatively in our clinic for a femur fracture. In addition, it was found that the patient was using 1000 mg of metformin per oral due to type 2 diabetes. At the preoperative cardiology evaluation, the ejection fraction was 60% with normal systolic ventricular function. Routine laboratory tests were normal. Metformin was held 24 hours before surgery. Spinal anesthesia was applied with 10 mg bupivacaine and 20 mcg fentanyl. Total blood loss at surgery was 150 cc. After an uneventful surgery, the patient was observed at the surgical postanesthesia care unit. Cardiac and respiratory physical examinations seemed normal but the patient had minimal acidosis and hypoxia in the arterial blood gas analysis. Twelve hours after the operation, compensated high anion gap ( 30 mEq/l) metabolic acidosis emerged, but lactate was normal. The patient's urea and creatinine levels were normal in the control blood tests, and the patient's urine output was above 0.5 ml/kg. Within this period, glucose levels were around 80-140 mg/dl. To overcome metabolic acidosis, bolus intravenous 8.4 % bicarbonate solution was administered. Bicarbonate infusion was started on the continuation of metabolic acidosis and base loss despite the bolus bicarbonate treatment. Since there was no other reason for the metabolic acidosis, metformin usage was considered to cause metabolic acidosis. During this treatment period, despite high anion gap acidosis, there was no lactate elevation. The patient had normal laboratory and hemodynamic values and was discharged from the intensive care unit at postoperative Day 3.

## Introduction

Metformin is a biguanide-derived drug commonly used in the treatment of type 2 diabetes since the 1960s. It acts by inhibiting glucose absorption from the intestine, reducing gluconeogenesis in the liver, and increasing the use of peripheral glucose [1]. As metformin is eliminated from the proximal tubules without metabolization, it may accumulate in the body during renal insufficiency. Most metformin-induced toxicities are seen due to acute or chronic renal diseases, advanced liver problems, or simultaneously occurring acute diseases [2].

The reported side effects of metformin are frequently associated with the gastrointestinal tract. However, metformin-induced metabolic acidosis, which is a serious complication with a mortality rate of 50%, may rarely occur. This complication is known to be associated with lactic acid elevation [3].

In this case report, we aimed to present the management of a patient who developed metabolic acidosis without lactic acidosis after metformin use in the intensive care unit.

## Case presentation

A 74-year-old male patient applied to the emergency department because of a femur fracture. He was admitted to the orthopedics and traumatology department with an operation plan. The comorbidities of the patient were type 2 diabetes mellitus, hypertension, chronic obstructive pulmonary disease, and benign prostatic hyperplasia. His medications were benidipine hydrochloride 8 mg, theophylline, tamsulosin HCl 0.4 mg, ipratropium bromide monohydrate + salbutamol sulfate 20/100 mcg, formoterol + budesonide 12/200 mcg, tiotropium bromide monohydrate 18 mcg, salbutamol inhaler 2.5 mg, and metformin 1000 mg twice a day.

In the preoperative cardiology examination, the ejection fraction of the patient was evaluated as 60% and the left ventricular systolic function was normal. The hemoglobin level was 10.4 g/dL in laboratory blood tests, and no other abnormality was detected. The metformin treatment was stopped 24 hours before the operation. In the operating room, the patient underwent spinal anesthesia with 10 mg bupivacaine and 20 mcg fentanyl. The surgery lasted for 90 minutes. The patient had 150 ml hemorrhage and received saline 1000 ml infusion with 500 ml polyglycine. Hemodynamic values were stable throughout the operation and the patient was taken to the intensive care unit (ICU) for postoperative observation. The cardiac and respiratory examination of the patient was repeated in the ICU. Mild acidosis and hypoxia were detected in the admission arterial blood gas (ABG) examination (Table 1). The patient's albumin level was 2 g/dL and human albumin 20% replacement treatment was applied. In the postoperative twelfth hour, metabolic acidosis with increased compensated anion clearance (30 mEq/L) was detected in the ABG analysis. The lactate level was within normal limits (Table 1). Urea and creatinine values were normal in the control blood tests and the hourly urine output of the patient was above 0.5 ml/kg. The hourly blood glucose measurements were between 80 and 140 mg/dL within the first 12 hours. Since a hemoglobin level of 8.4g/dL was detected, the patient received a unit of erythrocyte suspension. Although a bolus dose of bicarbonate 8.4% was administered intravenously, metabolic acidosis and the severe base deficit of the patient did not improve. So, intravenous bicarbonate infusion was initiated. As no additional reason for the persistent metabolic acidosis was detected, metformin was thought to be the cause of the metabolic situation of the patient.

At the postoperative twenty-fourth hour, the patients' metabolic acidosis was resolved and the bicarbonate infusion was terminated. Although there was high anion-gap (AG) metabolic acidosis, the lactate level of the patient was within normal ranges during this period. The patient was discharged from the ICU on the postoperative third day.

Table 1 shows the ABG values after admission to the ICU.

**Table 1:** ABG values after admission to the ICU ABG: arterial blood gas; ICU: intensive care unit

Arterial blood gas analysis	Admission values	12^th ^hour values
pH	7.33	7.34
PaCO_2 _(mmHg)	39	21
PaO_2 _(mmHg)	55	98
SaO_2_	89 %	97 %
HCO_3_(mmol/L )	20.6	11.3
Lactate (mmol/L )	1.5	0.7
Chlorine (mmol/L )	107.5	102.3

Sodium	136	138

## Discussion

Acid-base disorders are a commonly seen problem in the ICU. When the metabolic status of the patients is assessed in the ICU, it is seen that only 35% have a normal acid-base balance. Metabolic acidosis occurs either by the accumulation of a constant acid in the blood or by the loss of blood bases. In order to distinguish these reasons, the term AG was defined.

The AG value is the difference between the total major cations and anions in the plasma [4]. The most common cause of high AG metabolic acidosis is lactic acidosis. In addition, hypoxia, high doses of aspirin, methanol, ethylene glycol, paraldehyde, toluene, azotemic renal failure, ethanol, liver failure, ketoacids, fasting, and diabetes can also cause metabolic acidosis with high AG [5]. Despite the increased AG, the lactate levels of our patient ranged between 0.5 and 1.5 mmol/L. 

Our patient was slightly hypoxic at admission to the ICU, however, mild acidosis and hypoxia were detected in the admission arterial blood gas (ABG) examination. At the twelfth hour of intensive care, metabolic acidosis progressed despite heating and fluid replacement. So, we ruled out hypoxia from the etiology of metabolic acidosis. The use of metformin was considered the most likely cause of acidosis; hence, the patient did not have known aspirin or substance use or renal or hepatic insufficiency and had normal blood glucose levels. However, urinary output and urea creatinine levels were normal.

Although the most common side effects of metformin use are gastrointestinal problems, such as nausea and vomiting; hyperlactatemia and metabolic acidosis are rare but potentially serious side effects that can cause mortality [6]. These risks are thought to increase in cases of kidney or liver dysfunction, chronic heart or respiratory failure, sepsis and severe dehydration [7].

Metformin-associated lactic acidosis (MALA) is a rare but very dangerous complication of metformin use [8]. The presence of co-morbidities that may cause lactic acidosis is the most important factor that makes the differential diagnosis difficult. A meta-analysis by Inzucchi and colleagues reported a strong association between metformin and MALA development in patients with stable chronic kidney disease. MALA often develops in the case of concomitant acute disease, which can lead to acute renal failure [9].

However, so far, there is no case report regarding metabolic acidosis without lactic acidosis due to metformin use in the current literature. Despite the presence of type 2 diabetes in our patient, there was no kidney or liver failure and no increase in laboratory lactate levels.

In the case of MALA, the most important step in the treatment is ensuring adequate intravenous hydration. In addition, the use of targeting oxygen-sensing prolyl hydroxylase domain enzyme (PHD) has recently been proposed. Oyaizu-Toramaru and colleagues reported that the inhibition of PHD activates the transcription factor called hypoxia-inducible factor (HIF), which increases lactate excretion and gluconeogenesis in which lactate is used as a substrate [10]. Angioi et al. have reported that they applied continuous low-efficiency dialysis and bicarbonate to 28 patients with MALA and got positive results. We didn’t consider dialysis in the treatment of our patient because lactic acid levels were within the normal ranges. However, bicarbonate infusion was applied to correct the resistant acidosis.

In our patient, the termination of metformin use, increasing hemoglobin levels above 10 g/dL to improve oxygen delivery, and bicarbonate infusion for 24 hours successfully treated the metabolic status.

## Conclusions

Metformin-induced metabolic acidosis with increased lactic acidosis is a well-known complication of metformin treatment, with a high mortality rate. In addition, clinicians should also consider that metformin-induced metabolic acidosis may develop without lactate elevation. In such cases, metformin treatment should be terminated and bicarbonate infusion should be initiated.
